# Defining Veteran Rurality: An Analysis of the U.S. Veterans Health Administration Rural–Urban Taxonomy

**DOI:** 10.1111/jrh.70182

**Published:** 2026-07-10

**Authors:** Diana J. Govier, Travis I. Lovejoy, Sarah S. Ono, Eric R. Litt, Lauren K. Wilson, Zachary Burningham, Alexandra B. Caloudas, Peter J. Kaboli, M. Bryant Howren

**Affiliations:** ^1^ Veterans Rural Health Resource Center‐Portland VHA Office of Rural Health Portland Oregon USA; ^2^ Center to Improve Veteran Involvement in Care VA Portland Health Care System Portland Oregon USA; ^3^ School of Public Health Oregon Health & Science University and Portland State University Portland Oregon USA; ^4^ Department of Psychiatry Oregon Health & Science University Portland Oregon USA; ^5^ North Florida‐South Georgia Veterans Health Care System Gainesville Florida USA; ^6^ Salt Lake City Veterans Affairs Medical Center Salt Lake City Utah USA; ^7^ Division of Epidemiology, Department of Internal Medicine University of Utah Salt Lake City Utah USA; ^8^ Veterans Rural Health Resource Center‐Salt Lake City VHA Office of Rural Health Salt Lake City Utah USA; ^9^ Center For Innovations in Quality Safety and Effectiveness & South‐Central Mental Illness Research, Education and Clinical Center, Michael E. DeBakey VA Medical Center Houston Texas USA; ^10^ Menninger Department of Psychiatry & Behavioral Sciences Baylor College of Medicine Houston Texas USA; ^11^ Veterans Rural Health Resource Center‐Iowa City VHA Office of Rural Health Iowa City USA; ^12^ Department of Internal Medicine University of Iowa Carver College of Medicine Iowa City USA; ^13^ Center For Comprehensive Access and Delivery Research and Evaluation Iowa City Veterans Affairs Healthcare System Iowa City USA; ^14^ Holden Comprehensive Cancer Center University of Iowa Iowa City USA

**Keywords:** access to care, drive time, rural–urban commuting areas, veteran, veterans health administration

## Abstract

**Purpose:**

Rurality is a key consideration for the Veterans Health Administration (VHA) in improving access to care. However, VHA's rural–urban taxonomy, which seeks to guide these efforts, has not been evaluated since new VHA geographic access standards were implemented. Therefore, we examined the degree to which VHA's and University of Washington's “Categorization B” rural–urban taxonomies designate the same veterans as rural and compared drive times to VHA care based on these taxonomies.

**Methods:**

We determined Rural Urban Commuting Area (RUCA) codes of census tracts of VHA enrollees in Fiscal Year 2023 and rural–urban designations under VHA and Categorization B taxonomies, which each have three designations: urban/rural/highly rural and urban/large rural/small rural, respectively. For each RUCA code and rural–urban designation, we calculated frequencies and percentages of enrollees overall, and percentages of enrollees with drive times >30 minutes to VHA primary care sites and >60 minutes to VHA secondary and tertiary care sites, which parallel drive‐time eligibility standards for VHA‐purchased care under the 2018 Maintaining Internal Systems and Strengthening Integrated Outside Networks (MISSION) Act.

**Findings:**

Most (81.0%) enrollees had similar rural–urban designations under VHA and Categorization B taxonomies; divergence was primarily due to RUCA 2.0, which is considered rural by VHA but urban by Categorization B. Compared with the Categorization B large and small rural designations, the VHA rural and highly rural designations captured more veterans with drive times that meet eligibility standards for VHA‐purchased care under the MISSION Act. For example, 85.5% of veterans with tertiary care drive times >60 minutes were considered rural/highly rural by VHA while only 59.8% with tertiary care drive times of this length were considered large/small rural by Categorization B.

**Conclusions:**

Aligning with VHA's access‐to‐care priorities, VHA rural and highly rural designations better capture veterans who may experience geographic barriers to care. For other VHA access priorities, such as temporal access, integrating additional data may be necessary to refine rural‐urban taxonomies.

## 1 Introduction

Rurality is a key consideration for the Veterans Health Administration (VHA) in designing policies and directing resources to improve veteran health [1]. To guide these efforts, VHA has adopted a rural–urban taxonomy that employs Rural–Urban Commuting Area (RUCA) codes [2] to designate areas and veterans who reside within them as either urban, rural, or highly rural [3, 4], a taxonomy which was chosen, in part, to address VHA access‐to‐care policy priorities including standards for geographic access to care [5].

Compared with other federal taxonomies, VHA's rural designation is more broadly defined and its urban and highly rural designations are more narrowly defined. Prior research identified both advantages and drawbacks of VHA's designations for its planning and policy purposes [6]. Namely, compared with other federal taxonomies, actionable insights were clearer for VHA's narrowly defined highly rural veterans, the majority of whom faced long drive times to VHA care, whereas planning and policy implications were more opaque for VHA's broadly defined and dispersed rural veterans whose drive times varied. However, since VHA's taxonomy was last evaluated in 2010, new VHA geographic access standards were codified into law under the 2018 Maintaining Internal Systems and Strengthening Integrated Outside Networks (MISSION) Act [7].

Therefore, we sought to provide an updated examination of VHA's rural–urban taxonomy with respect to new MISSION Act VHA standards for geographic access to care, making use of VHA census tract data, which offer several advantages over the ZIP code data used in prior research [6, 8, 9]. To accomplish this, we compared the number of VHA enrollees and their drive times to VHA care sites based on VHA's taxonomy and the closest known alternative—“Categorization B,” which was developed by the University of Washington Rural Health Research Center, and, like VHA's taxonomy, assigns census tracts RUCA codes which are then aggregated into three rural–urban designations [2, 10].

## 2 Methods

### 2.1 Data Acquisition for Participants

VHA's Geospatial Service Support Center (GSSC) develops and maintains geographic data on all VHA enrollees and VHA sites of care, including geo‐coded physical addresses for enrollees and care sites, estimated drive times from enrollees’ physical addresses to VHA care sites, and RUCA code assignments at the census tract level. We obtained these data for all VHA enrollees with census tracts matching RUCA codes (*N* = 7,023,714) in Fiscal Year (FY) 2023 (i.e., October 1, 2022–September 30, 2023). Although not all VHA enrollees are active users of VHA care, we included all VHA enrollees because VHA is still responsible for their geographic access should they need/request care. We excluded individuals with RUCA code 99 (i.e., tracts with zero population) and/or VA priority group assignments rendering them ineligible for VHA health care benefits (*n* = 828,355), resulting in a sample of 6,195,651 VHA enrollees, which is roughly the size of the VHA enrollee population who used VHA care in FY2023 (*n* = 6,058,477) [11]. This work was characterized as pertinent to program operations for VHA's Office of Rural Health (ORH) and classified as a non‐research operational analysis. Thus, institutional review board evaluation was not required.

### 2.2 Study Variables

***VHA care sites***. VHA operates a variety of health care sites with site type determined based on criteria outlined in VHA Handbook 1006.02 [12]. “Tertiary care sites” are hospitals providing acute inpatient and/or rehabilitation services and with level 1 or 2 intensive care units (ICUs) and at least one of three complex clinical programs (cardiac surgery, complex neurosurgery, transplantation). “Secondary care sites” are either hospitals without level 1 or 2 ICUs and/or any of the three complex clinical programs, or sites which provide outpatient primary care, mental health care, specialty care, and ambulatory surgery. “Primary care sites” provide at least 500 annual outpatient primary care and/or mental health care encounters. Tertiary and secondary care sites are mutually exclusive site types; primary care sites are not mutually exclusive (e.g., a tertiary care site can also be a primary care site).

***Drive times***. The GSSC geo‐codes the home street addresses of VHA enrollees, combining latitudes and longitudes with travel time and road condition data to predict veterans’ average drive times, in minutes at 10 am on Wednesdays, to the nearest VHA tertiary care, secondary care, and primary care sites [6].

***RUCA codes***. The most commonly used method to classify US census tracts as rural or urban is the RUCA system [2]. The RUCA system comprises primary and secondary RUCA codes [2]. Primary RUCA codes define metropolitan and non‐metropolitan areas based on the size and direction of primary commuting flows. Secondary RUCA codes incorporate secondary or other connections among rural and urban areas. The two‐level structure facilitates flexible aggregation of codes to fit users’ needs. For this analysis, we used secondary RUCA codes available in GSSC files, which are derived from 2010 US Census data.

***Rural‐urban taxonomies***. VHA aggregates secondary RUCA codes into three designations for its rural–urban taxonomy: urban, rural, and highly rural (Table 1). Categorization B also has three designations based on secondary RUCA codes: urban, large rural city/town (hereafter “large rural”), and small and isolated rural town (hereafter “small rural”) [2]. Categorization B, which is the closest known alternative to VHA's taxonomy, has been used to study a variety of health care‐related topics [13, 14, 15, 16, 17]. For this analysis, we considered urban (Categorization B and VHA), large rural (Categorization B) and rural (VHA), and small rural (Categorization B) and highly rural (VHA) to be analogous designations.

**TABLE 1:** Number and percentage of enrollees based on Categorization B and VHA taxonomies.

	Urban (Cat B) Urban (VHA)	Large rural (Cat B) Rural (VHA)	Small rural (Cat B) Highly rural (VHA)
**Number of enrollees**			
Categorization B	4,978,959	649,072	567,620
VHA	4,126,490	1,827,071	242,090
**Percent of enrollees**			
Categorization B	80.4%	10.5%	9.2%
VHA	66.6%	29.5%	3.9%

Abbreviations: Cat B, Categorization B; VHA, Veterans Health Administration.

### 2.3 Analyses

We described the number and percentage of VHA enrollees based on each RUCA code and VHA and Categorization B rural–urban designations; mean drive times to VHA tertiary care, secondary care, and primary care sites based on each RUCA code; and the percentage of enrollees with drive times >30 minutes to primary care sites and >60 minutes to secondary care and tertiary care sites based on each taxonomy and designation. These thresholds were chosen to parallel VHA standards for geographic access to care set forth by the 2018 MISSION Act, which, along with other standards, determine eligibility for veterans to receive care purchased by VHA but furnished by non‐VHA providers (“VHA‐purchased care”).

## 3 Results

Overall, 66.6%, 29.5%, and 3.9% of VHA enrollees, respectively, resided in areas designated as urban, rural, and highly rural under VHA's taxonomy, while 80.4%, 10.5%, and 9.2% of enrollees, respectively, resided in areas designated as urban, large rural, and small rural under Categorization B (Table 1).

There was substantial (81.0%) overlap in the percentage of VHA enrollees who resided in areas with analogous designations based on VHA and Categorization B taxonomies (Table 2). For example, most enrollees (66.6%) resided in areas assigned RUCA code 1.0 or 1.1, which are designated as urban areas under both taxonomies. In addition, approximately one in ten (10.5%) resided in areas assigned RUCA code 4.0, 5.0, or 6.0, which are designated as rural or large rural areas under VHA and Categorization B taxonomies, respectively. Another 3.9% resided in areas assigned RUCA code 10.0, which, under VHA and Categorization B taxonomies, is considered a highly rural or small rural area, respectively.

**TABLE 2:** Rates and counts of VHA enrollees across rural–urban designations under Categorization B and VHA taxonomies.

RUCA code	RUCA code description	Categorization B designation	VHA designation	# VHA enrollees	% VHA enrollees
1.0	Metropolitan core (metro urban area): primary flow within urban area of ≥50,000 population	Urban	Urban	4,027,427	65.0%
1.1	1.0 + secondary flow 30%–50% to larger urban area	Urban	Urban	99,063	1.6%
2.0	Metropolitan high commuting: primary flow ≥30% to metro urban area	Urban	Rural	717,597	11.6%
2.1	2.0 + secondary flow 30%–50% to larger urban area	Urban	Rural	8,540	0.1%
3.0	Metropolitan low commuting: primary flow 10%–30% to metro urban area	Urban	Rural	62,009	1.0%
4.0	Micropolitan core (micro urban area): primary flow within urban area of 10,000–49,999 population	Large rural	Rural	399,580	6.5%
4.1	4.0 + secondary flow 30%–50% to metro urban area	Urban	Rural	34,421	0.6%
5.0	Micropolitan high commuting: primary flow ≥30% to micro urban area	Large rural	Rural	206,002	3.3%
5.1	5.0 + secondary flow 30–50% to metro urban area	Urban	Rural	4,672	0.1%
6.0	Micropolitan low commuting: primary flow 10%–30% to micro urban area	Large rural	Rural	43,490	0.7%
7.0	Small town core (small town urban area): primary flow within urban area of up to 9,999 population	Small rural	Rural	191,040	3.1%
7.1	7.0 + secondary flow 30%–50% to metro urban area	Urban	Rural	15,066	0.2%
7.2	7.0 + secondary flow 30%–50% to micro urban area	Small rural	Rural	5,370	0.1%
8.0	Small town high commuting: primary flow ≥30% to small town urban area	Small rural	Rural	78,856	1.3%
8.1	8.0 + secondary flow 30%–50% to metro urban area	Urban	Rural	1,803	0.03%
8.2	8.0 + secondary flow 30%–50% to micro urban area	Small rural	Rural	1,115	0.02%
9.0	Small town low commuting: primary flow 10%–30% to small town urban area	Small rural	Rural	32,669	0.5%
10.0	Rural area: primary flow to tract outside urban area	Small rural	Highly rural	242,090	3.9%
10.1	10.0 + secondary flow 30%–50% to metro urban area	Urban	Rural	8,361	0.1%
10.2	10.0 + secondary flow 30%–50% to micro urban area	Small rural	Rural	9,055	0.2%
10.3	10.0 + secondary flow 30%–50% to small town urban area	Small rural	Rural	7,425	0.1%

*Notes*: Shading of rural–urban designations is used to emphasize areas of concurrence and divergence.

Abbreviations: metro, metropolitan; micro, micropolitan; VHA, Veterans Health Administration.

The two taxonomies diverged for 19.0% of enrollees. Most of this divergence (13.7%) was due to Categorization B designations of urban that VHA designates as rural (RUCA 2.0, 2.1, 3.0, 4.1, 5.1, 7.1, 8.1, 10.1), with the preponderance of this difference (11.6%) accounted for by RUCA code 2.0. The Categorization B taxonomy employs a more liberal designation of small rural, which includes RUCA codes 7.0, 7.2, 8.0, 8.2, 9.0, 10.2, and 10.3, all of which the VHA taxonomy designates as rural. This accounted for the remaining 5.3% of the difference observed between taxonomies.

Mean drive times to VHA sites varied based on RUCA codes (Table 3). In general, as RUCA codes transition from more to less urban, mean drive times to tertiary, secondary, and primary care sites increased. Notably, mean drive times for certain RUCA codes that are considered urban under Categorization B, including 2.0, 2.1, 3.0, 4.1, 5.1, 7.1, 8.1, and 10.1, more closely resembled drive times for other RUCA codes considered large and small rural under Categorization B (4.0, 5.0, 6.0). For example, RUCA code 8.1 is considered urban under Categorization B, but has a mean drive time of 45 minutes for primary care and 168 minutes for tertiary care, which are similar to mean drive times for small rural designations (e.g., RUCAs 8.0, 9.0) under Categorization B.

**TABLE 3:** Mean drive times to VHA sites based on RUCA codes.

RUCA code	Categorization B designation	VHA designation	Mean drive time (in minutes) to tertiary care sites	Mean drive time (in minutes) to secondary care sites	Mean drive time (in minutes) to primary care sites
1.0	Urban	Urban	72	33	15
1.1	Urban	Urban	66	39	15
2.0	Urban	Rural	100	53	27
2.1	Urban	Rural	85	45	26
3.0	Urban	Rural	105	62	35
4.0	Large rural	Rural	135	76	29
4.1	Urban	Rural	104	57	24
5.0	Large rural	Rural	139	79	33
5.1	Urban	Rural	104	56	29
6.0	Large rural	Rural	113	68	34
7.0	Small rural	Rural	144	85	44
7.1	Urban	Rural	115	64	31
7.2	Small rural	Rural	170	119	31
8.0	Small rural	Rural	156	96	46
8.1	Urban	Rural	168	105	45
8.2	Small rural	Rural	112	81	43
9.0	Small rural	Rural	125	76	40
10.0	Small rural	Highly rural	166	96	54
10.1	Urban	Rural	163	82	40
10.2	Small rural	Rural	194	109	55
10.3	Small rural	Rural	205	123	68

*Notes*: Shading of rows is used to emphasize drive times considered “urban” under the Categorization B taxonomy but “rural” under the VHA taxonomy.

Abbreviations: VHA, Veterans Health Administration.

**FIGURE 1:**
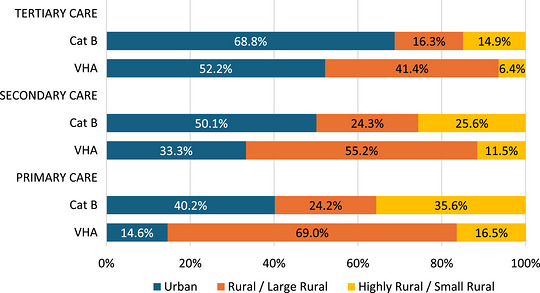
Percentage of enrollees with drive times exceeding 60 minutes to VHA tertiary or secondary care sites and 30 minutes to VHA primary care sites under Categorization B and VHA taxonomies. Denominator for tertiary care = 3,489,303 enrollees with drive times >60 minutes to tertiary care; denominator for secondary care = 1,604,982 enrollees with drive time >60 minutes to secondary care; denominator for primary care = 1,198,403 enrollees with drive times >30 minutes to primary care. Cat B, Categorization B; VHA, Veterans Health Administration.

Figure 1 characterizes the percentage of VHA enrollees who met drive time thresholds that parallel drive time eligibility standards for VHA‐purchased care under the MISSION Act. Compared with Categorization B designations of large and small rural, VHA's designations of rural and highly rural capture a greater percentage of veterans who had tertiary care, secondary care, and primary care drive times that parallel drive time eligibility standards for VHA‐purchased care. For example, 59.8% of veterans with primary care drive times >30 minutes were considered large or small rural by Categorization B, while 85.5% of veterans with primary care drive times >30 minutes were considered rural or highly rural by VHA. Under Categorization B, a greater percentage of urban veterans met drive time thresholds that parallel these eligibility standards. For example, 68.8% of veterans with tertiary care drive times >60 minutes were considered urban by Categorization B, while 52.2% of veterans with tertiary drive times >60 minutes were considered urban by VHA.

## 4 Discussion

Our findings indicate that relative to a frequently cited rural–urban taxonomy that closely parallels VHA's taxonomy (Categorization B), VHA's taxonomy more accurately captures rural‐residing veterans with drive times that may indicate suboptimal access to care and who may require attention from VHA to enhance access. Indeed, a “jump” in drive time was observed from RUCA code 1.1 to 2.0, which represents the transition from urban to rural in VHA's taxonomy. VHA's definitions of rural and highly rural thus serve a purpose of identifying enrollees whose geographic distance from VHA care would make them eligible for VHA‐purchased care through the MISSION Act, potentially reducing their travel burden to receive care.

This is not to say that VHA's rural–urban taxonomy is most suitable for addressing all VHA access‐to‐care policy priorities. For example, another criterion for VHA‐purchased care through the MISSION Act includes standards for temporal access (i.e., appointment wait times). Analysis of 2018 data showed that for some services (e.g., cardiology), rurality was associated with longer appointment wait times, while for others (e.g., orthopedics), rurality was not associated with differences in appointment wait times [18]. This suggests that VHA's taxonomy may need to be combined with other data, such as health professional shortage area data, if the goal is to understand and address VHA temporal access‐to‐care priorities. Overall, research is needed on relationships between VHA's rural‐urban taxonomy and other access‐to‐care policy priorities, which may help refine VHA's taxonomy and provide actionable insights for addressing these priorities.

### 4.1 Limitations

This study has limitations. Notably, the GSSC geo‐coded enrollee file that was available at the time of this analysis contained data from FY2023. Veterans who have newly enrolled in VHA since the release of this file are not included in our analysis. Second, this GSSC file uses RUCA 2010 codes. In July 2025, RUCA 2020 was made available, which is based on newly released 2020 Census population estimates and commuting flows [2]. At the time of our analysis, however, VHA had not updated the GSSC file to align with RUCA 2020—in a system as complex as VHA, updates across information systems take time to implement. Third, to calculate average drive times to VHA sites, VHA uses geographic information systems, which may not account for all real‐world road and driving conditions or changes to these that may impact drive times. Further, we used drive times to VHA sites as proxies for care access. Other factors influence access that were not considered in this study, such as need for services, availability and mode of transportation, income, health literacy, and cultural beliefs [19, 20, 21, 22]. Finally, given our study was based on VHA enrollees, VHA care sites, and VHA geographic access standards, our results may not generalize beyond this context. Nevertheless, our findings may offer broader insights for understanding rurality and its relationship with geographic access to care.

### 4.2 Conclusions and Future Directions

This work highlights the importance of how rurality is defined when assessing veterans’ geographic access to care and allocating VHA resources. Although both VHA and Categorization B taxonomies are derived from RUCA codes, their classifications yield meaningful differences in the identification of rural veterans and, consequently, who may be eligible for VHA‐purchased care under the MISSION Act. The VHA rural and highly rural designations better capture veterans with longer drive times—those most likely to experience geographic barriers to care—thereby aligning with the agency's geographic access‐to‐care policy objectives. Future research should explore how integrating additional data may refine rural–urban taxonomies to more accurately represent other priorities and dimensions of access. Continued collaboration among researchers and policymakers is essential to ensure that rural definitions effectively guide equitable health care access for all veterans.

## Funding

This work was supported by the VHA Office of Rural Health Career Development Award: NOMAD PROJ‐04269 (Govier & Caloudas), US Department of Veterans Affairs Career Development Award: IK2RD000662 (Govier), VHA Office of Rural Health: NOMAD PROG‐0000095 (Lovejoy & Ono) and NOMAD PROG‐0000042 (Howren). At the time of this study, all authors had employment or compensation arrangements wtih the US Department of Veterans Affairs.

## Disclosures

All statements in this manuscript, including its findings and conclusions, are solely those of the authors and do not necessarily represent the views of the US Department of Veterans Affairs, US government, Oregon Health & Science University, Portland State University, University of Utah, Baylor College of Medicine, or University of Iowa.

## Conflicts of Interest

The authors declare no conflicts of interest.

## Data Availability

The data that support the findings of this study may be available on request from the corresponding author. The data are not publicly available due to privacy or ethical restrictions.
